# Changes in P300 following alternate nostril yoga breathing and breath awareness

**DOI:** 10.1186/1751-0759-7-11

**Published:** 2013-05-31

**Authors:** Shirley Telles, Nilkamal Singh, Raghuraj Puthige

**Affiliations:** 1 Patanjali Research Foundation, Haridwar, India; 2 Swami Vivekananda Yoga Research Foundation, Bangalore, India; 3 Patanjali Research Foundation, Patanjali Yogpeeth, Maharishi Dayanand Gram, Bahadrabad, Haridwar, Uttarakhand 249405, India

**Keywords:** Alternate nostril yoga breathing, P300, Breath awareness, Cognitive processes

## Abstract

This study assessed the effect of alternate nostril yoga breathing (*nadisuddhi pranayama*) on P300 auditory evoked potentials compared to a session of breath awareness of equal duration, in 20 male adult volunteers who had an experience of yoga breathing practices for more than three months. Peak amplitudes and peak latencies of the P300 were assessed before and after the respective sessions. There was a significant increase in the P300 peak amplitudes at Fz, Cz, and Pz and a significant decrease in the peak latency at Fz alone following alternate nostril yoga breathing. Following breath awareness there was a significant increase in the peak amplitude of P300 at Cz. This suggests that alternate nostril yoga breathing positively influences cognitive processes which are required for sustained attention at different scalp sites (frontal, vertex and parietal), whereas breath awareness brings about changes at the vertex alone.

## Introduction

The ancient Indian way of life called yoga places considerable importance on breathing correctly. In one of the yoga texts it is said 'When the breath is irregular, the mind also is unsteady, but when the breath is still, so is the mind, and life is prolonged, hence one should regulate the breath (*Hatha Yoga Pradipika*, Chapter 2: Verse 2) [1].

Several yoga breathing techniques involve regulating the breath frequency, rhythm, phase durations, as well as the nostril through which a practitioner breathes [2]. These techniques involve conscious control of the breath with awareness and precision, and are called *pranayamas*. One such technique involves breathing alternately through the left and right nostril. Traditionally this practice called *nadisuddhi pranayama* is believed to help reach a state of satisfaction and reduce mental unrest, as well as restore physical and mental balance [1]. There have been studies to assess the effects of this alternate nostril yoga breathing (ANYB) technique on specific physiological and cognitive functions.

Among physiological variables studied, the electroencephalogram (EEG) was recorded in eighteen participants [3]. There were two test periods of alternate nostril yoga breathing with 5-minutes between them. In the first test period inhalation was through the right nostril and exhalation through the left nostril. The order was reversed in the second test period. In both test periods there was an increase in the mean power in the beta and alpha band, while in the second test period hemispheric asymmetry in the beta 1 band reduced, suggesting that alternate nostril yoga breathing has a balancing effect on the activity of the two cerebral hemispheres.

The effects of alternate nostril yoga breathing on tasks requiring hemisphere-specific skills did not show balancing, but showed lateralized effects, in a study on 135 children randomized to 5 groups (left uninostril breathing, right uninostril breathing, alternate nostril yoga breathing, breath awareness, and control) [4]. Each group had 27 participants who practiced the respective breathing technique four times a day for ten days. All 5 groups were assessed on a verbal memory task (left hemisphere specific) and a visuo-spatial memory task (right hemisphere specific) at the beginning and end of the 10-day period. The alternate nostril yoga breathing group showed improved visuo-spatial but not verbal memory scores at the end of 10 days. Apart from performance in tasks which were hemisphere-specific, alternate nostril yoga breathing improved the performance in a task requiring attention in another study [5]. Twenty healthy male volunteers practiced alternate nostril yoga breathing for 20 minutes. Before and after the practice their performance on a letter cancellation task was assessed. Cancellation tasks require sustained attention, visual scanning, activation and inhibition of rapid responses, and motor speed [6]. The results cited above [5], suggest that alternate nostril yoga breathing can improve attention. Cancellation tasks assess attention based on performance. Attention and immediate memory processes can also be objectively assessed by the P300 auditory event related potential [7]. The P300 reflects the ability to sustain and shift attention while discriminating between stimuli which differ in a single aspect, in this case, the frequency of tones. Previously, forced uninostril breathing practices improved the performance in the P300 discrimination task with a reduction in latency, which is an index of stimulus processing speed [8]. High frequency yoga breathing at 120 breaths per minute was practiced for one minute by fifteen healthy male participants who showed a decrease in the P300 latency after the practice.

Other factors apart from yoga practice can change the P300. The effect of emotions on a respiratory related evoked potential was studied when healthy volunteers were presented with pictures of pleasant, neutral or unpleasant content [9]. When the participants viewed either affectively pleasant or unpleasant pictures the P300 amplitude of the RREP was reduced, suggesting that emotions (pleasant or unpleasant) reduce attentional resources which are available for processing afferent respiratory sensory signals.

The relevance of this study to the present study is that meditation was found to help in uncoupling viewing negative emotional images and affect when yoga practitioners/meditators performed an event-related, affective Stroop task [10,11]. The findings suggested that meditators were able to uncouple viewing negative emotional images and affect when there were competing cognitive demands, not during emotional processing *per se*. These findings cannot be directly extrapolated to yoga breathing, though it would be of interest to determine whether yoga breathing techniques can help in uncoupling of negative emotions, given the close connections between breathing and emotions [12]. However this was not the aim of the present study.

The present study aimed at comparing the effects of alternate nostril yoga breathing with breath awareness on the P300 task. Breath awareness was selected for comparison as it is a part of ANY Band by comparing the effects of the two practices, it would allow the effects of breath awareness to be 'subtracted' from those of ANYB. Hence the hypothesis was that ANYB would influence the performance in the P300 discrimination task, while breath awareness would have no effect.

## Methods

### Participants

Twenty male volunteers with ages ranging from 21 to 38 years (group M ± S.D., 27.0 ± 4.9 years) participated in the study. They were residing at a yoga center (Swami Vivekananda Yoga Research Foundation, Bangalore, India). Participants were recruited through flyers distributed in the center. The inclusion criteria were: (i) the participants had experience of alternate nostril yoga breathing for more than 3 months (mean experience ± S.D., 30.2 ± 24.4 months), (ii) male participants alone were studied as auditory evoked responses have been shown to vary with the phases of the menstrual cycle [13] and the P300 (evoked by visual stimuli) also varied with gender [14], and (iii) all of them were in normal health based on a routine clinical examination. Exclusion criteria included: (i) a history of smoking, (ii) respiratory ailments including nasopharyngeal abnormalities, (iii) taking medication or using other wellness strategies, and (iv) any impairment affecting attention. The variables to be recorded and the study design were described to the participants and their signed consent to participate in the study was obtained. The study was approved by the ethics committee of Patanjali Research Foundation.

### Design of the study

**Figure 1:**
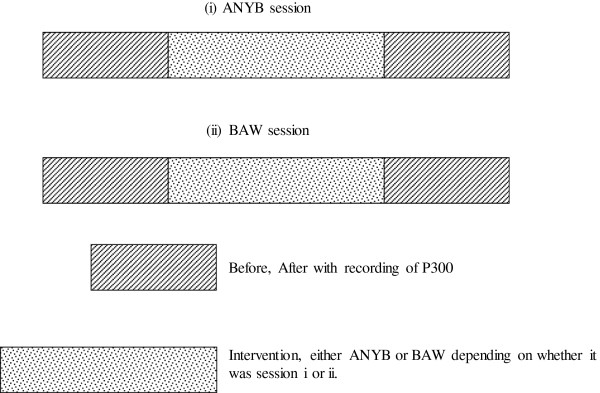
**Schematic representation of the design of the two sessions.** The P300 was recorded before and after the intervention. Periods of recording are shown as hatched and periods of intervention are shown as stippled.

Participants were assessed in two separate sessions namely, alternate nostril yoga breathing and breath awareness. For half the participants the alternate nostril yoga breathing session took place on the first day with breath awareness the next day. The remaining participants had the order of the sessions reversed. They were alternately allocated to either schedule to prevent the order of the sessions influencing the outcome. The participants were unaware about the hypothesis of the study. The assessments were done before and after each session which lasted for 40 minutes. The design has been shown schematically in Figure 1.

### Recording conditions

The peak latencies and peak amplitudes of P300 were recorded using Nicolet Bravo System (U.S.A.). The P300 component was elicited with a simple discrimination task known as the ‘oddball’ paradigm, in which two auditory stimuli are presented in a random series so that one of them occurs infrequently i.e., considered the oddball [7]. In our experience yoga practitioners found auditory stimuli less distracting than visual or somatosensory stimuli. For assessments participants were seated in a sound attenuated and dimly lit cabin and were monitored on a closed circuit television with instructions being given through an intercom, so that participants could remain undisturbed during a session.

### Electrode positions

Ag/AgCl disk electrodes were fixed with electrode gel (10–20 conductive EEG paste, D.O. Weaver, U.S.A.) at the Fz, Cz, and Pz scalp sites, with reference electrodes on linked earlobes and with the ground electrode on the forehead (FPz) according to the International 10–20 system [15]. The electro-ocular activity was recorded as an electro-occulogram (EOG), with a bipolar derivation from two electrodes placed 1 cm above and 1 cm below the outer canthus of the right eye to record the vertical EOG. The electrode impendence was kept below 5 kΩ at all sites.

### Amplifier settings

The electroencephalographic (EEG) activity was amplified with a sensitivity of 100 μV. The low cut filter was at 0.01 Hz and the high cut filter was at 30 Hz. P300 Event Related Potentials (ERPs) were computer averaged in 300 trial sweeps in the 0 to 750 ms range. The pre-stimulus delay was 75 ms and the level of artifact rejection was set at 90 percent.

### Stimulus characteristics

Binaural tone stimuli of alternating polarity delivered at 0.9 ms with a frequency of 1 KHz (50 cycles for the plateau, 10 cycles for the ramp) for the standard stimuli and 2 KHz (10 cycles for the plateau, 20 cycles for the ramp) for the target stimuli were used to trigger online averaging of the EEG. The percentage of standard stimuli was set at 80 and for the target stimuli at 20. The stimulus intensity was kept at 70 dB Sound Pressure Level (SPL). The inter stimulus interval was 1.1 ms.

### Recording procedure

Participants were asked to avoid substances which influence cognitive performance (particularly tea and coffee for the caffeine content) on the day preceding and on the day of the recording. Where this was unavoidable the session was taken on another day. The P300 evoked potentials were recorded with eyes closed and participants seated comfortably. The ‘standard’ and ‘target’ auditory stimuli were delivered through close fitting earphones (TDH-39, Amplivox, UK). Participants were asked to distinguish between the two tones and mentally count the ‘target’ stimuli. The equipment gives the number of target stimuli delivered. Only those sessions in which the participants achieved 95 percent accuracy in counting target stimuli were included. None of the sessions had to be excluded for this reason. The P300 responses were recorded before and immediately after the intervention.

### Interventions

#### Alternate nostril yoga breathing

Alternate nostril yoga breathing (ANYB) practice involves breathing through left and right nostrils alternately [16]. In this nostril manipulating *pranayama* the thumb and the ring finger of the right hand were used to manipulate or occlude the nostrils. This is a characteristic yoga gesture (*nasika mudra* in Sanskrit) prescribed during *pranayama* practice to manipulate the nostrils with ease [2]. Throughout this practice the awareness is directed to the breath and breathing.

#### Breath awareness

During breath awareness (BAW), the participants maintained awareness of the breath without manipulation of the nostrils. During these practices the participants’ attention was directed to the movement of air into and out of their nostrils. They also attempted to be aware of the air as it moved through the nasal passage.

### Data extraction

The peak amplitude and the peak latency of the P300 were measured at three electrode sites; i.e., Fz, Cz and Pz. The peak amplitude (in μV) was defined as the voltage difference between baseline at stimulus delivery and the largest positive-going peak of the ERP waveform within 250–500 ms latency [7]. The peak latency (ms) was defined as the time from stimulus onset to the point of maximum positive amplitude within the latency window (i.e., 250-500 ms).

### Data analysis

Statistical analysis was done using SPSS (Version 10.0). Data were analyzed using the repeated measures analyses of variance (ANOVA). There were separate ANOVAs for the peak amplitudes at the three sites (Fz, Cz and Pz) and for the peak latencies at the three sites (Fz, Cz and Pz). Hence there were six ANOVAs. Each ANOVA had two Within subjects factors. These were Sessions (ANYB, BAW) and States (Before, After).

*Post*-*hoc* tests with Bonferroni adjustment for multiple comparisons were used to detect significant differences between mean values, for pre-post comparisons.

## Results

For the peak amplitudes at Fz the repeated measures ANOVA showed a significant difference between the States (*F* = 11.67, *df* = 1,19, *P* < .01) and the interaction between Sessions and States (*F* = 7.63, *df* = 1,19, *P* < .01), suggesting the two were not independent of each other. For the peak amplitude the repeated measures ANOVA showed a significant difference between the States at Cz (*F* = 20.87, *df* = 1,19, *P* < .001) and Pz (*F* = 19.72, *df* = 1,19, *P* < .001).

Also, for the peak latency at Fz the repeated measures ANOVA showed a significant difference between the States (*F* = 5.02, *df* = 1,19, *P* < .05) and the interaction between Sessions and States (*F* = 5.38, *df* = 1,19, *P* < .05), suggesting the two were not independent of each other. For all comparisons the Hyunh-Feldt ϵ was equal to 1.00, hence sphericity was assumed.

**Figure 2:**
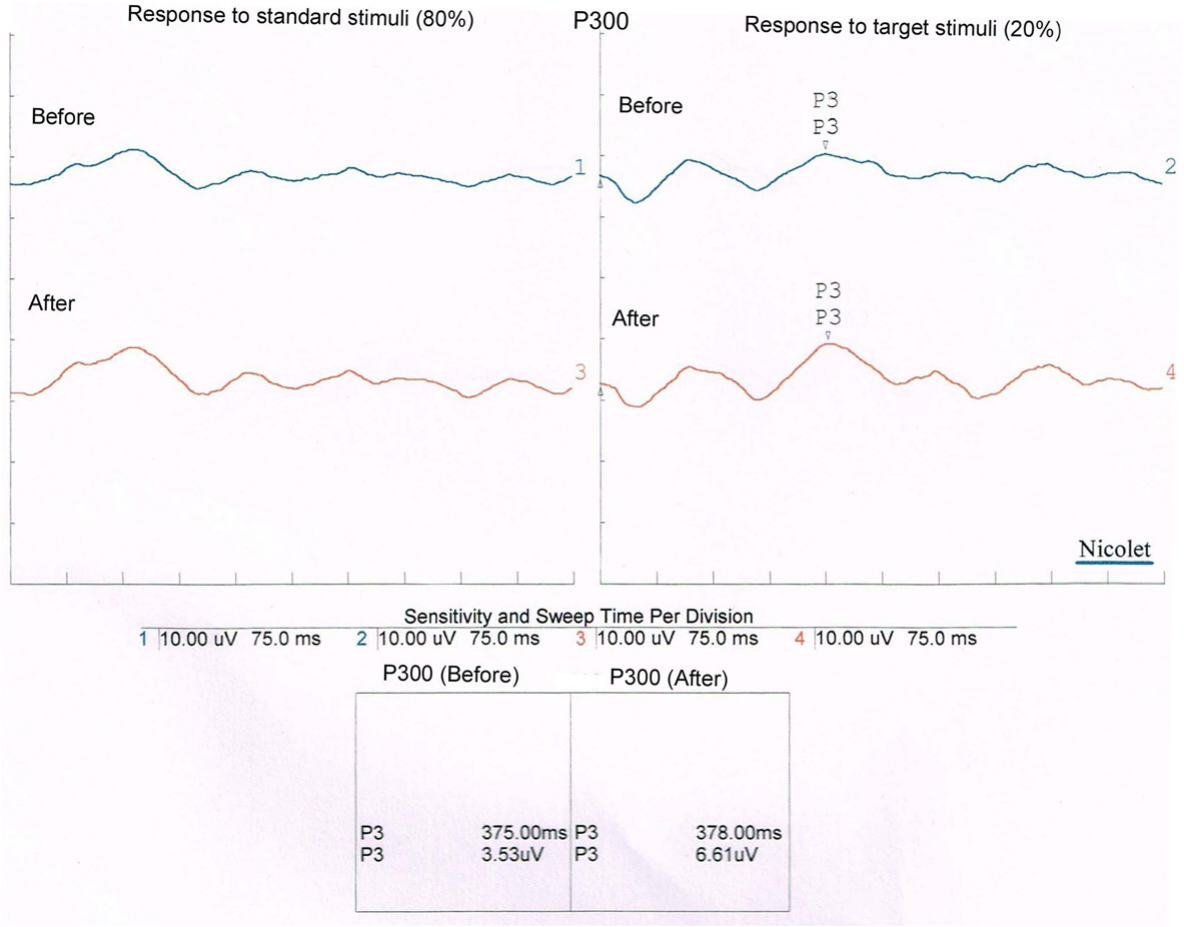
**A single sample of a P300 waveform.** The traces on the right show the responses to target stimuli before (2) and after (4) ANYB, showing an increase in P3 amplitude after ANYB (4) compared to before (2). Traces on the left show the response to standard stimuli before (1) and after (3) ANYB, with no change.

After the two sessions the following changes were seen. There was a significant increase in the P300 peak amplitude at Fz, Cz, and Pz sites after the practice of ANYB compared to before (*P* < .001). Also, there was a significant increase in the P300 peak amplitude at Cz after the practice of breath awareness compared to before (*P* < .05). However, there were no other significant changes following breath awareness. There was a significant decrease in the P300 peak latency at Fz site after the practice of ANYB compared to before (*P* < .05). However, there were no changes in peak latencies found at Cz, and Pz sites and also following breath awareness practice. The mean ± S.D. values of amplitude and latency at Fz, Cz and Pz electrode sites, before and after ANYB and BAW are provided in Table 1. A sample P300 waveform is shown in Figure 2.

**Table 1:** Peak amplitude (μV) and peak latencies (ms) of the P300 component in ‘pre’ and ‘post’ alternate nostril yoga breathing (ANYB) and breath awareness (BAW) (n = 20)

**Electrode site**^**†**^	**Variables**	**ANYB**			**BAW**		
		**Pre**	**Post**	**Cohen's *d***	**Pre**	**Post**	**Cohen's *d***
Fz	Amplitude (μV)	4.14 ± 2.81	7.37*** ± 4.82	0.85	4.36 ± 3.12	5.52 ± 3.84	0.33
	Latency (ms)	355.75 ± 26.26	336.65* ± 27.82	0.72	351 ± 28.43	352.75 ± 20.82	0.07
Cz	Amplitude (μV)	6.22 ± 5.59	10.06*** ± 6.21	0.65	6.55 ± 5.27	8.82* ± 5.10	0.44
	Latency (ms)	359.85 ± 33.34	352.65 ± 26.18	0.24	356.55 ± 25.54	353.70 ± 31.57	0.10
Pz	Amplitude (μV)	8.11 ± 4.96	11.12*** ± 5.37	0.58	8.55 ± 6.38	10.62 ± 5.52	0.35
	Latency (ms)	355.80 ± 32.02	346.95 ± 28.61	0.29	359.70 ± 20.09	354.30 ± 31.81	0.21

***P < .001, **P < .01, *P < .05 Bonferroni adjustment ‘Post’ compared with respective ‘Pre’ values^**†**^ Reference: Linked ear lobes.

Values are group mean ± SD.

*Post*-*hoc* tests for multiple comparisons were performed with Bonferroni adjustment. All comparisons were made with respective ‘pre’ states. The Cohen's *d* values were calculated and are provided in Table 1 for ANYB and BAW, amplitudes and latencies at the three sites (Fz, Cz and Pz).

## Discussion

Alternate nostril yoga breathing influenced the amplitudes of P300 at the vertex and at frontal and parietal sites, whereas breath awareness had lesser effects on the amplitudes of P300, seen at the vertex alone. P300 latency decreased after ANYB at Fz. The P300 amplitude is believed to indicate the resources available to process information about the stimuli [17]. The P300 latency reflects the speed of stimulus classification; it is generally not related to the overt response and is independent of the behavioral reaction time. The P300 latency is an index of stimulus processing rather than response generation and hence it is used as a motor-free measure of cognitive function. The P300 peak latency has been negatively correlated with mental functions in normal persons; shorter latencies are associated with superior cognitive performance in tasks for attention and immediate memory.

The increased P300 peak amplitude and latency following ANYB suggests that this practice increases the attentional resources along with better stimulus processing speed and efficiency.

The P300 can be influenced by several factors, including emotions. A decreased P300 amplitude in participants elicited while participants looked at emotionally charged images (compared to those which were neutral) [10] suggests that emotional responses can engage neural responses involved in eliciting the P300 response. There is a close connection between respiration and the emotions. For example, by breathing in a particular pattern which was characterized by the experimenters it was found that participants found it easier to simulate specific emotions, such as joy-laughter, sadness-crying, fear-anxiety, anger, erotic love and tenderness [18]. It is possible that yoga breathing practices reduce emotional responses. Alternate nostril yoga breathing was shown to reduce autonomic arousal in terms of lower systolic and diastolic blood pressure and reduced cutaneous vasoconstriction [19]. With lower levels of physiological arousal participants may have been more relaxed, and hence they may have had more attentional resources available for the P300 task. However no autonomic variables were measured in this study and this remains a speculation.

The increase in amplitude in the P300 at Fz following breath awareness supports earlier research that this practice can improve performance in tasks requiring attention [20]. Maximum changes in the P300 peak amplitude occurred at Fz (Cohen's *d* = 0.85) compared to Cz (Cohen's *d* =0.65) and Pz (Cohen's *d* = 0.58). Also the P300 peak latency decreased at Fz alone. While a previous P300 study [21] and neuroimaging studies [22] have shown frontal changes in meditators, the difference between the P300 amplitudes recorded at Fz, Cz and Pz is not enough to conclude that the changes occurred in frontal areas, as other factors contribute to the difference.

In summary the present results suggest that alternate nostril yoga breathing can increase the resources required to perform the P300 task. These findings are compatible with reports of better cancellation task scores after ANYB [20]. The results may be due to lower anxiety, as anxiety is associated with a general inability to maintain an attentional focus [23].

The findings are limited by the following factors (i) a small sample size, (ii) absence of a no intervention control group, (iii) the fact that assessments immediately followed the practice does not allow any conclusion to be drawn about how long the effects last, and (iv) there were no additional variables recorded, such as the state anxiety or autonomic variables and affective valence, which could have helped in understanding mechanisms underlying the changes seen.

Despite these limitations the results suggest an application of ANYB in improving the ability to attend to and discriminate between auditory stimuli.

## Competing interests

The authors declare that they have no competing interests.

## Authors’ contributions

ST designed the study, interpreted the results and compiled the manuscript. NS assissted in data analysis, interpretation and compiling the manuscript. RP collected the data, analyzed and interpreted it and assisted in compiling the manuscript. All authors read and approved the final manuscript.
